# Coherent states and unilateral coherence in strengthening entropic uncertainty relations

**DOI:** 10.1038/s41598-025-95598-3

**Published:** 2025-04-12

**Authors:** Forough Panahyazdan, Ahmad Akhound

**Affiliations:** Department of Physics, Payame Noor University, P.O. Box 19395-3697, Tehran, Islamic Republic of Iran

**Keywords:** Entropic uncertainty relation, Heisenberg uncertainty principle, Entanglement, Coherence state, Physics, Information theory and computation, Quantum physics

## Abstract

In this paper, we examine entropy uncertainty relations in the presence of quantum memory and quantum entanglement for non-orthogonal states constructed from two-qubit coherent states. Our results reveal that for the studied state, the entropy uncertainty relations under quantum memory conditions remain tight across a broad range of parameters, leading to lower error rates and higher precision in observable predictions. We explicitly demonstrate that in the presence of quantum memory, the ability to predict observables is fundamentally tied to entanglement. Stronger entanglement leads to reduced entropy uncertainty and greater predictive accuracy. On the other hand, as entanglement weakens, even though the uncertainty relations remain tight, prediction errors increase accordingly. In the maximum entanglement state, the accuracy of the predictions is also maximized. As the entanglement decreases, despite the tightening of the uncertainty relations, the accuracy of the predictions decreases. The prediction accuracy is fully proportional to the amount of entanglement, and it is possible to achieve the desired minimum entropy uncertainty and high correlation according to the problem’s requirements. This is while the numerical values of the components forming the upper bound of the entropy uncertainty relations are not equal in all cases. With respect to the changes examined from the presented state, quantum correlation, entropy uncertainty, and the components forming the upper bound, regardless of the amount of entanglement, each separately tend to a constant value.

## Introduction

Uncertainty is one of the most fundamental principles governing the quantum world, first presented in 1927 by Heisenberg^1^. The general concept of this principle indicates that the exact value of a pair of incompatible observables, such as *x* and *y*, cannot be determined simultaneously, and the measurement of each one leads to uncertainty in determining the exact amount resulting from the observation. It is no longer acceptable and leads to the limitation of simultaneous measurement of incompatible pairs. Uncertainty is expressed in terms of the commutation relationship $\left[X,Y\right]=XY-YX$ and $\Delta {\text{X}}^{2}=\langle {X}^{2}\rangle -\langle X\rangle { }^{2}$^2,3^.

1 $${\Delta X}^{ }{\Delta Y}^{ }\ge \frac{1}{2}\left|\langle \left[X,Y\right]\rangle \right|,$$

Shannon entropy plays a fundamental role in quantum information theory, analogous to variance in classical uncertainty relations. The entropic uncertainty relation introduced by Maassen and Uffink^4^ expresses Heisenberg’s uncertainty principle in terms of Shannon entropy and is given by:

2 $$H\left(X\right)+H\left(Z\right)\ge {\text{log}}_{2}\frac{1}{\text{c}},$$

where $H\left(X\right)=-\sum_{\text{i}=1}^{\text{d}}{p\left({x}_{i}\right)}_{ }{\text{log}} \, p\left({x}_{i}\right)$ denotes the Shannon entropy of the measurement outcomes in basis $X$, and ${c}_{ij}={max}_{ij}{\left|\langle {x}_{i}\left|{z}_{j}\right.\rangle \right|}^{2}$ quantifies the maximum overlap between the two measurement bases $X$ and $Z.$

Studying entropic uncertainty relations in the presence of quantum memory and quantum entanglement is crucial for understanding the fundamental limits of quantum information processing. These relations determine the fundamental constraints on the precision of predicting measurement outcomes of non-commuting observables, which play a key role in quantum cryptography, quantum metrology, and quantum error correction. In the presence of quantum memory, this relationship was investigated in terms of von Neumann’s conditional entropy $S\left(A|B\right)=S\left({\rho }_{AB}\right)-S\left({\rho }_{B}\right)$ and was introduced by Berta^5^ as follows:

3 $$S\left(X|B\right)+S\left(Z|B\right)\ge {\text{log}}_{2}\frac{1}{c}+S\left(A|B\right),$$

In this relation, $S\left(\rho \right)=-\sum_{m}{\rho }_{m}{\text{log}}{\rho }_{m}$ where ${\rho }_{m}$ are the eigenvectors of $\rho$ is the von Neumann entropy. ${\rho }_{B}$ is the reduced density of subsystem $B$ (quantum memory). Another relationship was presented by Adabi^6^ as follows:

4 $$S\left(X|B\right)+S\left(Z|B\right)\ge {\text{log}}_{2}\frac{1}{c}+S\left(A\left|B\right.\right)+max\left\{0.\delta \right\},$$

In this relation, the parameter *δ* is defined as $\delta =I\left(A;B\right)-\left[I\left(X;B\right)+I\left(Z;B\right)\right]$. Relationship (4) is tighter and more comprehensive than relationship (3). In this article, the left boundaries of the two inequalities (3) and (4) are called ${U}_{l}$, the right boundary of relation (3) is called ${U}_{r}^{b}$, and the right boundary of relation (4) is called ${U}_{r}^{a}$.

Quantum coherence represents the non-orthogonal space of the system. One of the properties of quantum coherent states is the minimization and, in other words, the tightness of the Heisenberg uncertainty relation for them. For a pair of incompatible observables $X$ and $P$, the uncertainty relation is expressed as ${\Delta X}^{ }{\Delta P}^{ }=\frac{1}{2}\left|\langle \left[X,P\right]\rangle \right|$ with ${\Delta X}^{ }={\Delta P}$^7,8^.

The coherence measure used in this work is defined as $C({\rho })$, which quantifies the amount of coherence in a given quantum state^9^. This function maps the density matrix to a real and non-negative value. In the case of a two-part system, quantum coherence can be analyzed based on the subsystems, known as a one-way quantum coherence process^10^. Additionally, the relationship of entropy uncertainty in the presence of quantum memory is described using relative coherence entropy^11^ for relations (3) and (4)^12^.

5 $${C}_{rel}^{X}\left({\rho }_{AB}\right)+{C}_{rel}^{Z}\left({\rho }_{AB}\right)=H\left(X|B\right)+H\left(Z\left|{B}\right.\right)-2S\left(A\left|{B}\right.\right)\ge {\text{log}}_{2}\frac{1}{c}-S\left(A|B\right),$$

And

6 $${C}_{rel}^{X}\left({\rho }_{AB}\right)+{C}_{rel}^{Z}\left({\rho }_{AB}\right)=H\left(X|B\right)+H\left(Z\left|{B}\right.\right)-2S\left(A\left|{B}\right.\right)\ge {\text{log}}_{2}\frac{1}{c}-S\left(A|B\right)+max\left\{0.\delta \right\}.$$

The relative coherence measure ${C}_{rel}^{X}\left({\rho }_{AB}\right)$ characterizes the coherence shared between subsystems A and B in the measurement basis *X*. The left side of these two inequalities is called* C* ${U}_{l}$, the right boundary of relation (5) is* C* ${U}_{r}^{b}$, and the right boundary of relation (6) is* C* ${U}_{r}^{a}$. Considering that relations (5) and (6) are based on the real and non-negative value of the system state, they reflect the mirror image of relations (3) and (4).

## Non-orthogonal two-qubit mode and quantum correlation

Coherent states are considered the quantum states closest to classical space, making their study and examination significant. The non-orthogonal state under consideration, which includes cohesive states, is introduced as follows^14^:

7 $$\left|\right.{\Phi }_{ } \rangle =\frac{1}{N} [u\left| \right.\xi \rangle \otimes \left| \right.1 \rangle +v\left| \right.\xi \rangle \otimes \left| \right.0 \rangle +w\left|\xi 1 \right.\rangle \otimes \left| \right.1 \rangle +z\left| \right.\xi 1 \rangle \otimes \left| \right.0 \rangle ],$$

the system includes coherent states $\left|\right.\xi \rangle$ and $\left|\right.\xi 1\rangle$, which can be measured as follows by defining them through orthogonal bases:

8 $$\left|\right.\xi \rangle =\frac{1}{\sqrt{1+{\left|\xi \right|}^{2}}}\left| \right.0 \rangle +\frac{\xi }{\sqrt{1+{\left|\xi \right|}^{2}}}\left| \right.1 \rangle ,\quad \left| \right.\xi 1 \rangle =\frac{1}{\sqrt{1+{\left|\xi 1 \right|}^{2}}}\left| \right.0 \rangle +\frac{\xi 1 }{\sqrt{1+{\left|\xi 1 \right|}^{2}}}\left| \right.1 \rangle ,$$

the normalization coefficient is obtained based on the situation mentioned as follows:

9 $${N}^{2}={\left|u\right|}^{2}+{\left|v\right|}^{2}+{\left|w\right|}^{2}+{\left|z\right|}^{2}+{u}^{*}w \langle \xi \left| \right.\xi 1\rangle +{v}^{*}z \langle \xi \left| \right.\xi 1\rangle +{w}^{*}u \langle \xi 1\left| \right.\xi \rangle +{z}^{*}v \langle \xi 1\left| \right.\xi \rangle .$$

In this relation, the inner product between the coherent state components is defined as $\langle \xi \left| \right.\xi 1\rangle =\frac{1+\overline{\xi } \xi 1}{\sqrt{(1+{\left|\xi \right|}^{2})(1+{\left|\xi 1\right|}^{2})}}$, where $\overline{\xi }$ is the complex conjugate of $\xi$. This inner product quantifies the overlap between the coherent states and directly influences the entropic uncertainty bounds.

Entanglement is considered one of the types of quantum correlations. By using relations (7) and (8), along with some calculations^14^, and defining the variables dependent on coherent states, ${\varvec{M}}=\sqrt{1-{\left|\langle \xi \left| \right.\xi 1\rangle \right|}^{2}}$ and ${\varvec{P}}=\langle \xi \left| \right.\xi 1\rangle$, for a two-qubit system in standard bases, we will have the following:

10 $$\left|\right.{\Phi } \rangle =\frac{1}{N} [z{\varvec{M}}\left| \right.00\rangle +w{\varvec{M}}\left| \right.01\rangle +(v+z{\varvec{P}})\left|10 \right.\rangle +(u+w{\varvec{P}})\left| \right.11\rangle ],$$

The normalization factor *N* will be as follows:

11 $${N}^{2}={\left|z{\varvec{M}}\right|}^{2}+{\left|w{\varvec{M}}\right|}^{2}+{\left|(v+z{\varvec{P}})\right|}^{2}+{\left|(u+w{\varvec{P}})\right|}^{2}.$$

The density matrix is ${\rho }=\left|\Phi \rangle \langle \Phi \right|$. The density matrix for the investigated state is 4 × 4 and the reduced density matrix are as ${\rho }_{1}={Tr}_{2}\left|\Phi \rangle \langle \Phi \right|$ and ${\rho }_{2}={Tr}_{1}\left|\Phi \rangle \langle \Phi \right|$ are calculated. The eigen values of the reduced density are obtained as follows:

12 $${\uplambda }_{\pm}=\frac{1}{2}\left(1\pm \sqrt{1-{\left({C}\left(\left| \right.{\Phi }_{\begin{array}{c} \\ \end{array}} \rangle \right)\right)}^{2}}\right),$$

For the mentioned state, the reduced density eigenvalues are obtained as follows:

13 $${\uplambda }_{\pm}=\frac{1}{2}\pm \frac{1}{2}\sqrt{1-4{\left|\frac{uz-vw}{{N}^{2}} \right|}^{2}{\left|{\varvec{M}} \right|}^{2}} ,$$

$C(|\Phi \rangle )$ represents the entanglement of the two-qubit system^13–16^. Entanglement is one of the fundamental properties of quantum systems, manifesting as a form of non-classical correlation between the subsystems of a composite system. For a two-qubit system, entanglement can be quantified using various measures, such as concurrence or von Neumann entropy. In this study, we define the entanglement of the investigated state using the following relation:

14 $$C(|\Phi \rangle ) =2{\left|\frac{(u\text{z}-vw){\varvec{M}}}{{N}^{2}}\right|}.$$

where the coefficients $u,\text{z},v and w$ and represent the parameters defining the state, ${\varvec{M}}$ quantifies the overlap between the coherent states, and $N$ is the normalization factor. This expression indicates that the degree of entanglement depends on the interplay between the state’s coefficients and the coherence overlap.

## Uncertainty and quantum correlation for a non-orthogonal state:

In recent years, entropic uncertainty relations have been examined for different states^15,17–24^. In our previous work, we investigated this relation for coherent states that were simultaneously entangled and squeezed spin^17^. In another previous study, we analyzed this relation for general two-qubit coherent and maximally entangled states, which could also be extended to other two-qubit states^18^. In this paper, we examine entropic uncertainty relations for the non-orthogonal state^14^, which includes coherent states.

15 $$|\Phi \rangle = \frac{1}{N} [\cos (\uptheta ){\text{e}}^{{{\text{i}}\varphi }} |\xi \rangle \otimes |1\rangle + \sin (\uptheta ){\text{e}}^{{{\text{i}}\varphi }} |\xi \rangle \otimes |0\rangle {}_{ + }^{ - } \sin (\uptheta ){\text{e}}^{{ - {\text{i}}\varphi }} |\xi 1\rangle \otimes |1\rangle {}_{ - }^{ + } \cos (\uptheta ){\text{e}}^{{ - {\text{i}}\varphi }} |\xi 1\rangle \otimes |0\rangle ],$$

In this relation $\left|w\right|=\mp \left|v\right|$, $\left|z\right|=\pm \left|u\right|$ with the following definitions:

16 $$u = \cos (\uptheta )e^{{i\upvarphi }} ,\quad v = ~\sin (\uptheta )e^{{i\upvarphi }} ,\quad w = {}_{ + }^{ - } ~\sin (\uptheta )e^{{ - i\upvarphi }} ,\quad z = {}_{ - }^{ + } ~\cos (\uptheta )e^{{ - i\upvarphi }} ,$$

If the state (15) is rewritten using relation (8) in the form of relation (10), the result will be as follows:

17 $$\begin{aligned} {{|\Phi }}\rangle & = \frac{1}{N}\left[ {\left( {{ }\left[ {\frac{v}{{\sqrt {1 + \left| \xi \right|_{ }^{2} } }} + \frac{z}{{\sqrt {1 + \left| {\xi 1} \right|_{ }^{2} } }}} \right]|00\rangle } \right) + \left( {\left[ {\frac{{{ }u}}{{\sqrt {1 + \left| \xi \right|_{ }^{2} } }} + \frac{w}{{\sqrt {1 + \left| {\xi 1} \right|_{ }^{2} } }}} \right]} \right)|01\rangle } \right. \\ & \quad \left. { + \left( {\left[ {\frac{{{ } v\xi }}{{\sqrt {1 + \left| \xi \right|_{ }^{2} } }} + \frac{z\xi 1}{{\sqrt {1 + \left| {\xi 1} \right|_{ }^{2} } }}} \right]} \right)|10\rangle + \left( {\left[ {\frac{{{ } u\xi }}{{\sqrt {1 + \left| \xi \right|_{ }^{2} } }} + \frac{w\xi 1}{{\sqrt {1 + \left| {\xi 1} \right|_{ }^{2} } }}} \right]} \right)|11\rangle ,} \right]{ } \\ \end{aligned}$$

The normalization coefficient will also be given by using relation (11) in the following form:

18 $$N = \sqrt {2\cos \left( \uptheta \right)^{2} + 2\sin \left( \uptheta \right)^{2} } ,$$

with the application of the angles θ = π/4 and φ = 0, the density matrix is derived as follows:

19 $$\rho _{{AB}} = \left( {\begin{array}{*{20}c} {\frac{1}{2}\left( U \right)_{~}^{2} } & {~\frac{1}{2}~\left( D \right)\left( U \right)} & {\frac{1}{2}~\left( U \right)\left( W \right)} & {\frac{1}{2}~\left( U \right)\left( Q \right)} \\ {\frac{1}{2}~\left( D \right)\left( U \right)} & {\frac{1}{2}\left( D \right)_{~}^{2} } & {\frac{1}{2}~\left( D \right)\left( W \right)} & {\frac{1}{2}~\left( D \right)\left( Q \right)} \\ {\frac{1}{2}~\left( U \right)\left( W \right)} & {~~\frac{1}{2}~\left( D \right)\left( W \right)} & {\frac{1}{2}\left( W \right)_{~}^{2} } & {\frac{1}{2}~\left( Q \right)\left( W \right)} \\ {\frac{1}{2}~\left( U \right)\left( Q \right)} & {\frac{1}{2}~\left( D \right)\left( Q \right)} & {\frac{1}{2}~\left( Q \right)\left( W \right)} & {\frac{1}{2}\left( Q \right)_{~}^{2} } \\ \end{array} } \right),$$

wherein the elements of the matrix ${\rho }_{AB}$ are as follows:

20 $$\begin{aligned} & U = \left( {\frac{1}{{\sqrt 2 \sqrt {1 + \left| {\xi } \right|_{ }^{2} } }} + \frac{1}{{\sqrt 2 \sqrt {1 + \left| {\xi 1 } \right|_{ }^{2} } }}} \right), \quad D = \left( {\frac{1}{{\sqrt 2 \sqrt {1 + \left| {\xi } \right|_{ }^{2} } }} - \frac{1}{{\sqrt 2 \sqrt {1 + \left| {\xi 1 } \right|_{ }^{2} } }}} \right), \\ & W = \left( {\frac{\xi }{{\sqrt 2 \sqrt {1 + \left| \xi \right|_{ }^{2} } }} + \frac{\xi 1}{{\sqrt 2 \sqrt {1 + \left| {\xi 1} \right|_{ }^{2} } }}} \right),\quad Q = \left( {\frac{\xi }{{\sqrt 2 \sqrt {1 + \left| \xi \right|_{ }^{2} } }} - \frac{\xi 1}{{\sqrt 2 \sqrt {1 + \left| {\xi 1 } \right|_{ }^{2} } }}} \right), \\ \end{aligned}$$

In this article, entanglement and entropic uncertainty relations in the presence of quantum memory on the non-orthogonal state composed of coherent states are examined. Three different data points, $\xi =1, 2, 3$ within the range $\xi 1= \mp \frac{1}{\xi }$ and the angles $\uptheta =\frac{\pi }{4}$ and ${\upvarphi }=0$, are the values on which the results are based. The observables of interest will be the Pauli matrices, $X={\sigma }_{x}$ and $Z={\sigma }_{z}$. Highlighting the key role of the entropic uncertainty relation and its importance in various fields of quantum information theory, we further raise the question of whether state (17) can be a good candidate for states that tighten the entropic uncertainty relation or not. To this end, we will also examine relation (3) in the following form:

21 $$\Delta_{xz}\, {: =}\, S\left( {X|B} \right) + S\left( {Z|B} \right) - S\left( {A|B} \right) - \log_{2} \frac{1}{c} \ge 0.$$

In the mentioned relation, ${\Delta }_{xz}$ represents the uncertainty in the simultaneous measurement of a pair of incompatible observables. It is also important to note that the equality sign in the above equation indicates the realization of the tight uncertainty condition. Next, we will analyze the graph of this relation.

**Fig. 1:**
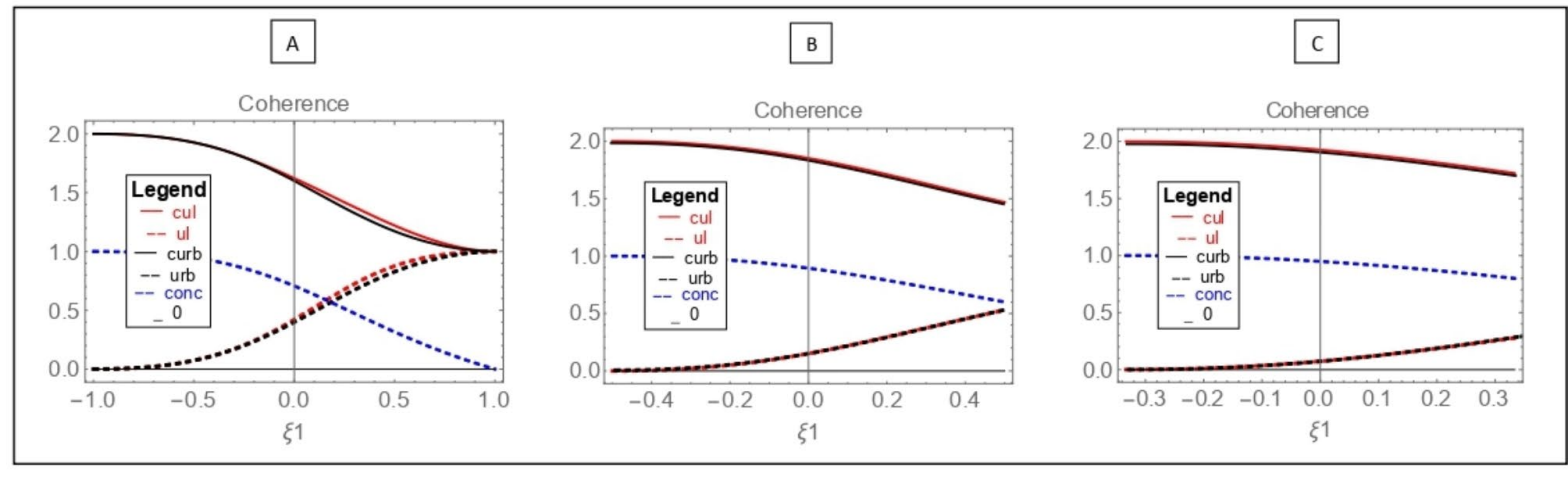
Entropic uncertainty relations in the range $\frac{1}{\xi }\ge \xi 1\ge -\frac{1}{\xi }$ according to the angles $\uptheta =\frac{\pi }{4}$,${\upvarphi }=0$. (**A**)$\upxi =1$, ${U}_{l}\cong {U}_{r}^{b}$ (red dashed line, black dashed line), $C{U}_{l}\cong C{U}_{r}^{b}$(red line, black line). (**B**) $\upxi =2$, ${U}_{l}={U}_{r}^{b}$, and $C{U}_{l}=C{U}_{r}^{b}$. (**C**) $\upxi =3$, ${U}_{l}={U}_{r}^{b}$, and $C{U}_{l}=C{U}_{r}^{b}$. entanglement (blue dashed line).

In Fig. 1, the left bound of relation (3) is shown by the ${U}_{l}$ graph (red dashed line), and the right bound of relation (3) is shown by the ${U}_{r}^{b}$ graph (black dashed line) for state (17) with different values of $\upxi$ from zero point.

Additionally, in Fig. 1, using the left bound of Eq. (5), the $C{U}_{l}$ (red line) chart and the right bound of Eq. (5), $C{U}_{r}^{b}$(black line) are drawn. Based on the results of the diagram (A), the sides of the entropy uncertainty relationships in the presence of quantum memory have been ized from the beginning of the charts and with an increase in $\xi 1$, the two bounds $C{U}_{l}$ and ${U}_{l}$ slightly deviate from their full match with the right bound *C* ${U}_{r}^{b}$ and ${U}_{r}^{b}$, and eventually at $\upxi 1=1$, all charts (3) and (5) coincide, producing the same result.

In the state (17) examined in the article, Eq. (5) behaves similarly to Eq. (3) in reverse. The entropy uncertainty relationships in a wide range of $\frac{1}{\xi }\ge \xi 1\ge -\frac{1}{\xi }$ for the state presented in the article are tight. Entanglement is also plotted using Eq. (14) (blue dotted line) from point one, the maximum value. The examined diagram shows that with an increase in $\upxi 1$, entanglement decreases and disappears at $\upxi 1=1$. In diagram (B), these points are also examined for $\upxi =2$. According to this diagram, the entropy uncertainty based on Eqs. (3) and (5) is tight in the entire range $\frac{1}{\xi }\ge \xi 1\ge -\frac{1}{\xi }$. Such a result follows the accuracy of predicting the results of the two components in ${U}_{l}$ and* C* ${U}_{l}$. This is while with the increase in $\upxi 1$, the entanglement decreases from its maximum value. It is obvious that at the maximum entanglement state, the accuracy of predicting the results under study will be maximized. With the reduction of entanglement, despite the tightening of uncertainty relations, the accuracy of the predictions will also decrease.

Accordingly, in diagram (C), the mentioned cases for $\upxi =3$ are also examined. According to this diagram, the entropy uncertainty relations in this state are also tight in all points similar to the $\upxi =2$ state, and the entanglement shows less decrease compared to the diagrams (A) and (B). Therefore, the reduction in the accuracy of predicting results in this state will also be less. By comparing all three diagrams, it can be concluded that changing the value of $\upxi$ affects the entropy uncertainty relationships and entanglement in such a way that increasing $\upxi$ tends to push each diagram towards the numerical value of the starting point of that diagram. Therefore, entanglement and the accurate prediction of entropy uncertainty results also increase. With the maximum amount of entanglement, the system will be free from the limitation of entropy uncertainty for predicting results, and the right bound of the entropy uncertainty relationship in the presence of memory will be zero.^5^ Such a result is provided for Eq. (5) with a numerical value of two for the right bound of the entropy uncertainty relationship.

**Fig. 2:**
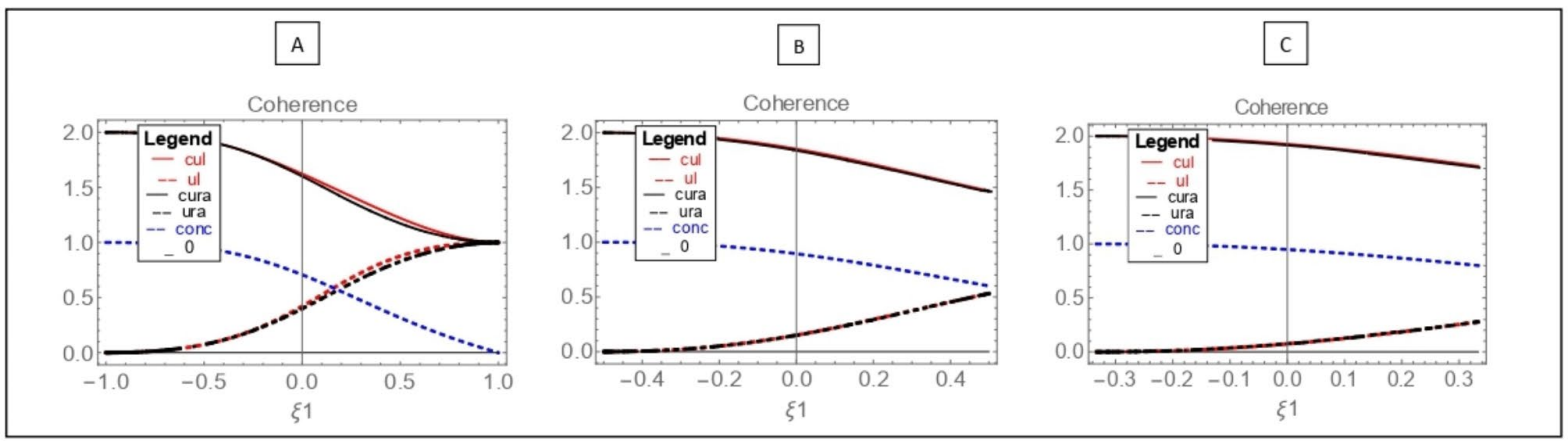
Entropic uncertainty relations in the range $\frac{1}{\xi }\ge \xi 1\ge -\frac{1}{\xi }$ according to the angles $\uptheta =\frac{\pi }{4}$,${\upvarphi }=0$. (**A**) $\upxi =1, {U}_{l}\cong {U}_{r}^{a}$ (red dotted line, black dotted line). $C{U}_{l}\cong C{U}_{r}^{a}$ (red line, black line). (**B**) $\upxi =2$,${U}_{l}={U}_{r}^{a}$ and $C{U}_{l}=C{U}_{r}^{a}$. (**C**) $\upxi =3$, ${U}_{l}={U}_{r}^{a}$ and $C{U}_{l}=C{U}_{r}^{a}$ entanglement (blue dotted line).

In Fig. 2, the left boundary of Eq. (4) is shown, similar to before, via the ${U}_{l}$ plot (red dashed line), and the right boundary of Eq. (4) with the ${U}_{r}^{a}$ plot (black dashed line) for the case (17) with different values of $\upxi$ from the zero point.

Additionally, in the plots of Fig. 2, using the left boundary of Eq. (6), the $C{U}_{l}$ plot (red line) and the right boundary of Eq. (6), the $C{U}_{r}^{a}$ plot (black line) are drawn. By comparing the plots of Figs. 1 and 2, it is evident that the behavior of Eq. (4) in Fig. 2, which is displayed with dashed lines, is exactly similar to the behavior of Eq. (3) in Fig. 1. Such behavior is also observed for the two Eqs. (6) in Fig. 2 and (5) in Fig. 1.

**Fig. 3:**
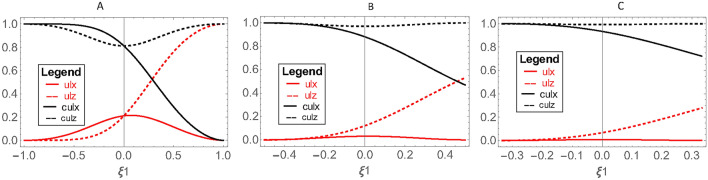
Components ${U}_{l}$, $S\left(X|B\right)\ne S\left(Z|B\right)$ (solid red line and red dashed line), components *C* ${U}_{l}$, ${{C}_{rel}^{z}\left({\rho }_{AB}\right)\ne C}_{rel}^{x}\left({\rho }_{AB}\right)$ (black dashed line and solid black line) as functions of the angles $\uptheta =\frac{\pi }{4}$, ${\upvarphi }=0$,$\frac{1}{\xi }\ge \xi 1\ge -\frac{1}{\xi }$. (**A**) $\upxi =1$. (**B**) $\upxi =2$. (**C**) $\upxi =3.$

The plots in Fig. 3 represent $S\left(X|B\right)$ (solid red line) and $S\left(Z|B\right)$ (red dashed line), which are components of the ${U}_{l}$ boundary, as well as ${C}_{rel}^{x}\left({\rho }_{AB}\right)$ (solid black line) and ${C}_{rel}^{z}\left({\rho }_{AB}\right)$ (black dashed line), which are components of the *C* ${U}_{l}$ boundary. Unlike the two components of the left boundary in the Heisenberg uncertainty relation that are equal in the coherent state, there is no such equality between the two components $S\left(X|B\right)$ and $S\left(Z|B\right)$ corresponding to the ${U}_{l}$ boundary, nor between ${C}_{rel}^{x}\left({\rho }_{AB}\right)$ and ${{C}_{rel}^{z}\left({\rho }_{AB}\right)}$, components of the *C *${U}_{l}$ boundary.

According to Fig. 3B, the two components $S\left(X|B\right)$ and $S\left(Z|B\right)$ of the ${U}_{l}$ boundary are initially equal, but as $\upxi 1$ increases, they exhibit differences. Similar conditions hold for the components of the *C* ${U}_{l}$ boundary, but in reverse. By comparing Fig. 3A–C, we observe that as $\upxi$ increases, the components of the uncertainty bound, $S\left(X|B\right)$ and $S\left(Z|B\right)$, approach each other in magnitude, indicating a trend towards reducing uncertainty. However, they remain distinct due to the inherent properties of the chosen quantum states. This behavior is also observed for the components of the *C* ${U}_{l}$ boundary,${C}_{rel}^{x}\left({\rho }_{AB}\right)$ and ${C}_{rel}^{z}\left({\rho }_{AB}\right)$.

**Fig. 4:**
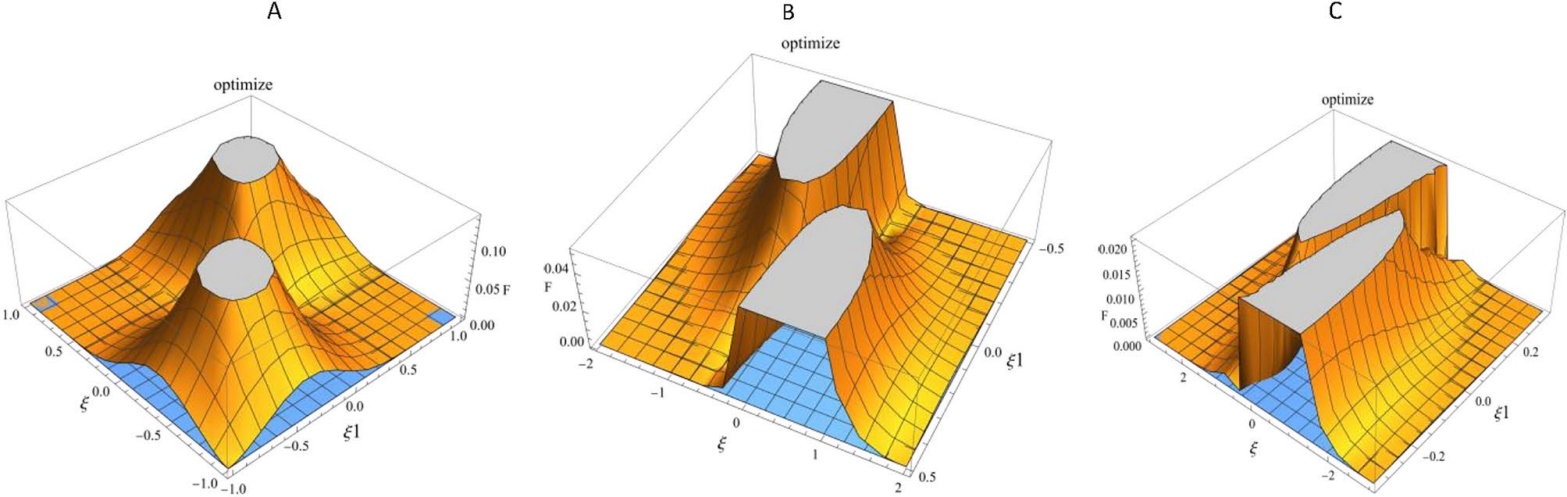
Three-dimensional plot of the entropy uncertainty ${\Delta }_{xz}$ as a function of the angles $\uptheta =\frac{\pi }{4}$, ${\upvarphi }=0$ and $\xi 1=\mp \frac{1}{\xi }$. (**A**) $\xi =\pm 1$. (**B**) $\xi =\pm 2$. (**C**) $\xi =\pm 3$.

In Fig. 4, the three-dimensional plot of entropy uncertainty ${\Delta }_{xz}$ as a function of the angles $\uptheta =\frac{\pi }{4}$, ${\upvarphi }=0$, over the range $\xi 1=\mp \frac{1}{\xi }$ and $\xi =$ 1, 2, 3 is drawn to examine the tight points, and it can be used according to the problem’s requirements.

**Fig. 5:**
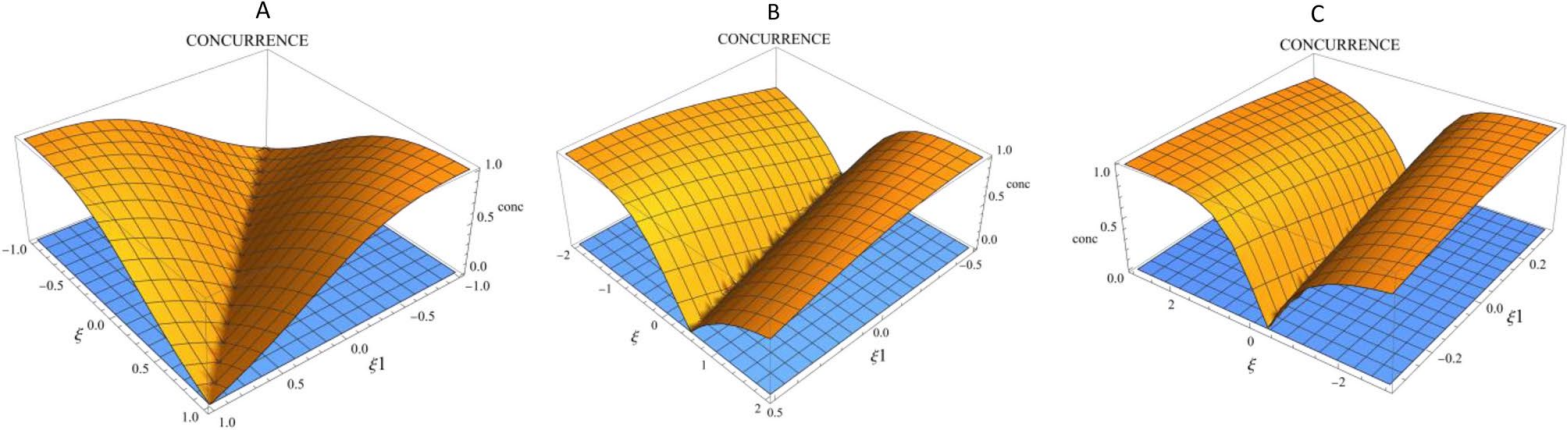
Three-dimensional plot of concurrence as a function of the angles $\uptheta =\frac{\pi }{4}$, ${\upvarphi }=0$, and $\xi 1=\mp \frac{1}{\xi }$. (**A**) $\xi =\pm 1$. (**B**) $\xi =\pm 2$. (**C**) $\xi =\pm 3$.

Similarly, in Fig. 5, the three-dimensional plot of entanglement as a function of the angles $\uptheta =\frac{\pi }{4}$, ${\upvarphi }=0$, over the range $\xi 1=\mp \frac{1}{\xi }$ and $\xi =$ 1, 2, 3 is depicted.

To optimize the general form of the ${\Delta }_{xz}$ relation, we reformulated the function in terms of parameters associated with the non-orthogonal coherent quantum state and employed numerical methods to determine its optimal value. This process involved exploring the parameter space and analyzing the function’s behavior to identify the optimal points.

### Optimal points

Numerical calculations indicate that the optimal value of is achieved at specific points in the parameter space. Specifically, the optimal parameters are:

- $$\xi$$ = 2.7148
- $$\xi 1$$ = 0.3683
- $$\uptheta$$ = 1.4022
- $${\upvarphi }$$ = 6.2831
- $${\Delta }_{xz}=0.0000$$

Note: The numerical values have been rounded to four decimal places for clarity. If higher precision is required, the exact values can be used accordingly.

At these values, ${\Delta }_{xz} \text{reaches}$ its minimum. These results suggest that the coherence and entanglement structure of the system play a fundamental role in determining the uncertainty bound.

## Conclusion

In this paper, the entropy uncertainty in the presence of quantum memory was investigated based on three different relations for the non-orthogonal state consisting of coherent states. Based on the extensive results examined, three of which are mentioned in the paper, the tightness of the entropy uncertainty relations in the presence of quantum memory (similar to the conditions governing Heisenberg’s uncertainty) is established over a wide range of parameters for the presented state.

Our findings indicate that quantum entanglement serves as a critical resource for reducing entropy uncertainty and improving prediction accuracy. Unlike classical correlations, which do not lead to such improvements, quantum entanglement ensures that the uncertainty bounds remain tight while simultaneously enhancing predictive precision. This suggests that highly entangled states are optimal candidates for applications requiring precise quantum measurements. As entanglement decreases, the uncertainty bounds remain valid, but the actual ability to predict observables deteriorates, confirming that prediction accuracy is proportional to the degree of entanglement. Such a result ensures the accuracy of the predicted outcomes of the two components in ${U}_{l}$ and* C* ${U}_{l}$. Given the greater comprehensiveness of coherent states compared to non-coherent states and the robustness of the entropy uncertainty relations for the state mentioned in the paper, this state, due to maintaining tightness and correlation over a wide range of parameters, is considered a suitable option for future studies in this field, with lower error rates and greater extensiveness.

In this paper, it was shown that the tightness of the relations over a wide range is maintained while the relations ${U}_{l}={U}_{r}^{b },{U}_{r}^{a}$ and $C{U}_{l}={CU}_{r}^{b },{CU}_{r}^{a}$ hold, indicating that at points with maximum entanglement, the highest accuracy in predicting the results is achieved. As the entanglement decreases, even though the relations are tight, the accuracy in predicting the results decreases. Evidently, at the maximum entanglement, the accuracy in predicting the results is at its highest. With the decrease in entanglement, despite the tightness of the uncertainty relations, the predictive accuracy will decrease, and the accuracy of predictions will be directly proportional to the amount of entanglement.

The left boundary components of the entropy uncertainty relations $S\left(X|B\right)$ and $S\left(Z|B\right)$ corresponding to the ${U}_{l}$ boundary and also between ${C}_{rel}^{x}\left({\rho }_{AB}\right)$ and ${{C}_{rel}^{z}\left({\rho }_{AB}\right)}$ on the state under examination (unlike the prevailing trend in Heisenberg’s uncertainty) are not equal under all conditions. Therefore, the accuracy of the components under examination in the left boundary relations in the presented state consisting of coherent states will not necessarily be the same and will tend towards equality with an increase in $\upxi$. An increase in $\upxi$ will also lead the boundaries of the entropy uncertainty relations towards the numerical value of the starting point of the same plot. Therefore, as the entanglement tends to its maximum possible value, the accuracy in predicting the results will also tend towards its maximum value. The optimization of the relation confirms that, for a specific set of parameters, the uncertainty can approach its minimal value. These findings provide valuable insights into the fundamental limits of quantum information and may have potential applications in quantum information processing and cryptography.

## Data Availability

The datasets used and/or analyzed during the current study are available from the corresponding author upon reasonable request.
